# Supplementary material for: The sands of time run faster near the end
Source: Nat Commun. 2017 Jun 1;8:15551. doi: 10.1038/ncomms15551 (PMC5461489; doi:10.1038/ncomms15551)
Supplement: Supplementary Information — Supplementary Figures and Supplementary Table [file ncomms15551-s1.pdf]

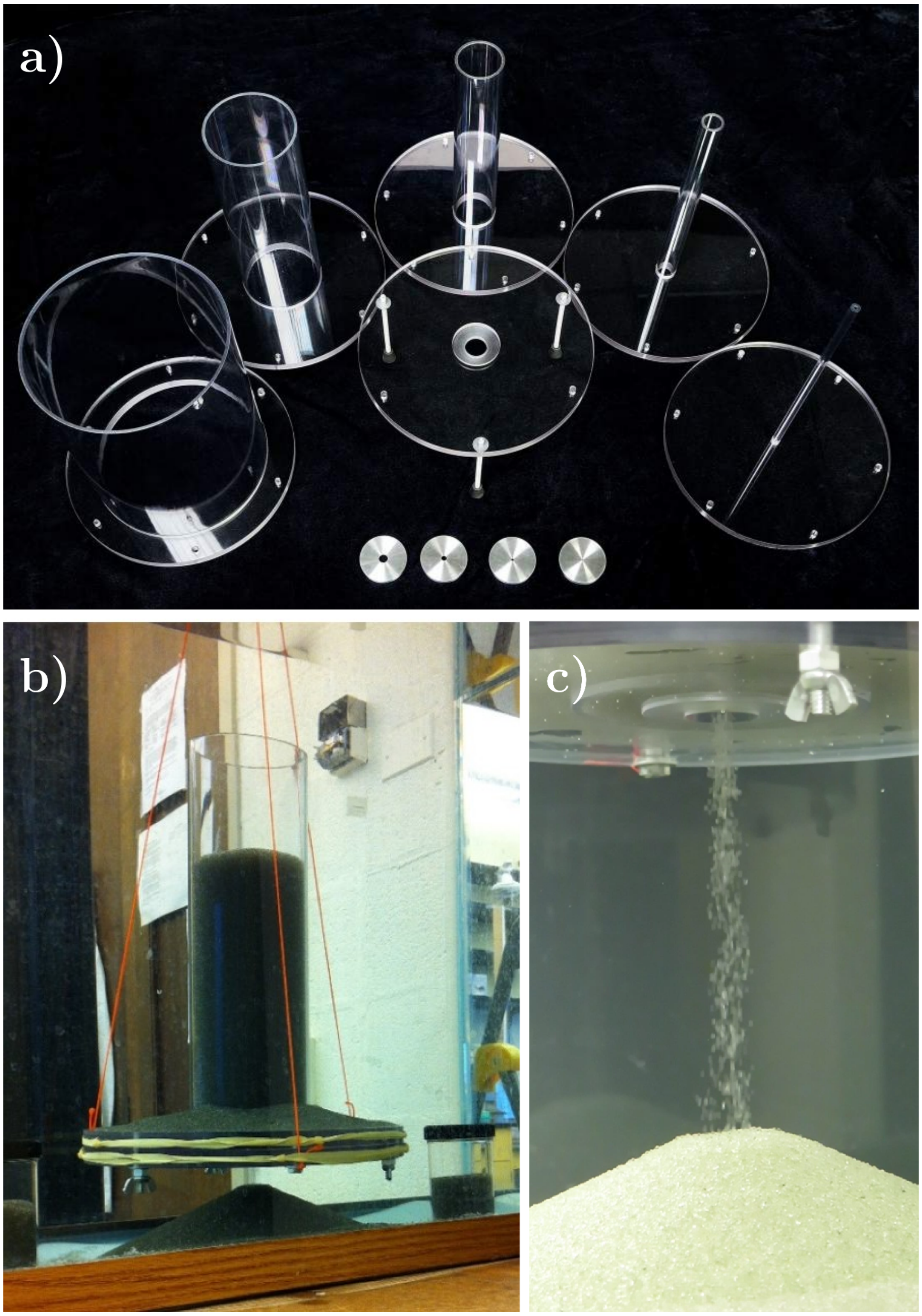

Supplemetary Figure 1: Photographs of the experimental device.
(a) The measurement device consists of interchangeable polycarbonate tubes and 5.1 cm diameter aluminum disks with concentric orifices that can be fitted into the depression in the polycarbonate bottom plate shown at the center of the figure. (b) In the submerged case the hopper is completely under water in a fish tank, so that water can freely flow in at the top as grains exit at the bottom. The orange strings connect the hopper to the digital scale. (c) The $d = 0.1$ cm grains flow in steady stream from the $D = 0.6$ cm orifice, fully submerged under water.

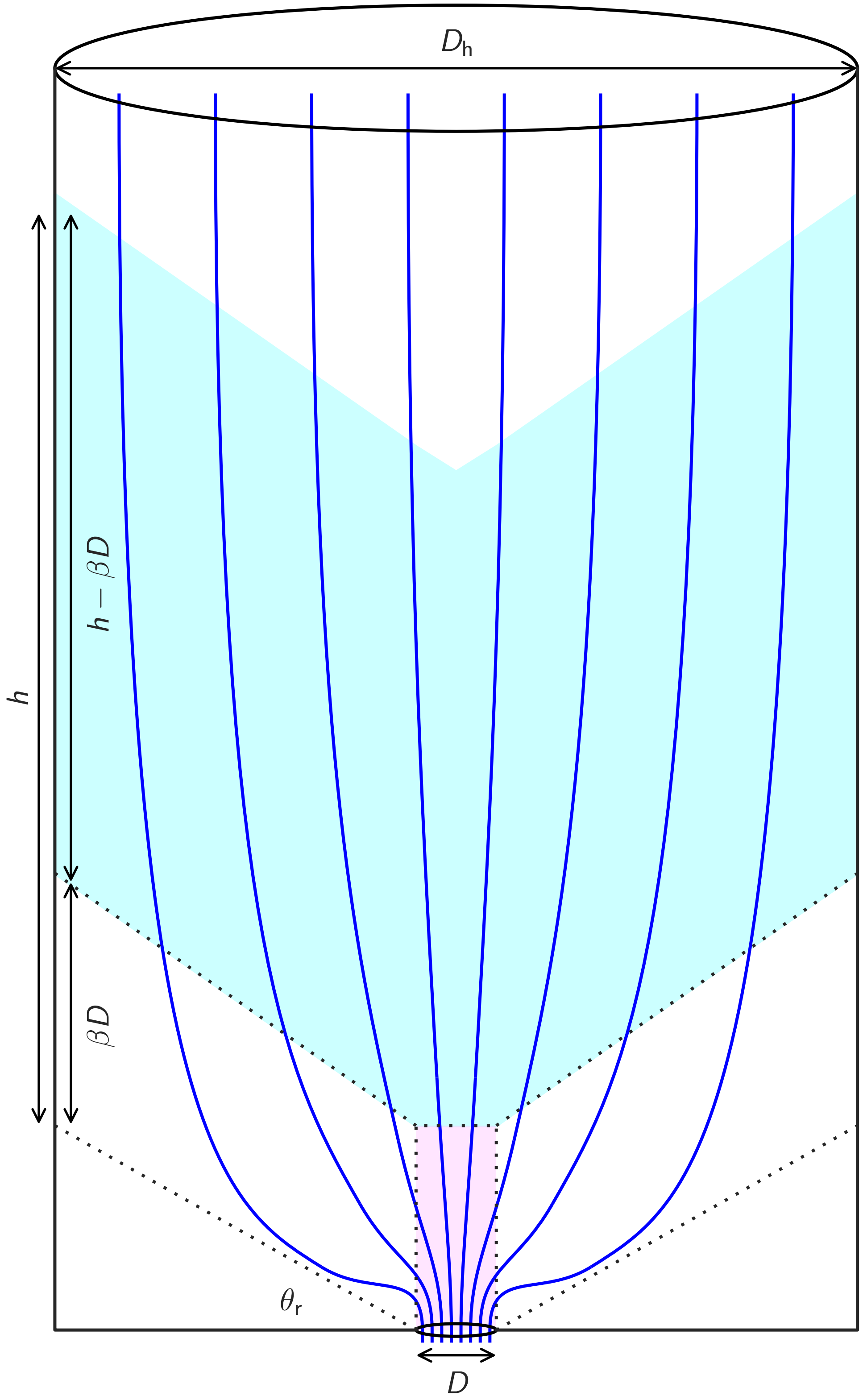

Supplemetary Figure 2: Schematic illustration of the open experiment. Schematic illustration of the open experiment, similar to Fig. 1b of the main text. The light blue and red shaded areas represent the two regions of the porous granular medium as defined by the hole diameter D, the parameter β, and the height h of grains yet to be discharged. The dark blue curves represent possible streamlines for the flow of fluid through the medium. The angle of repose θ_r determines the set of grains that remain in the hopper after flow ceases, i.e. when h decreases to zero.

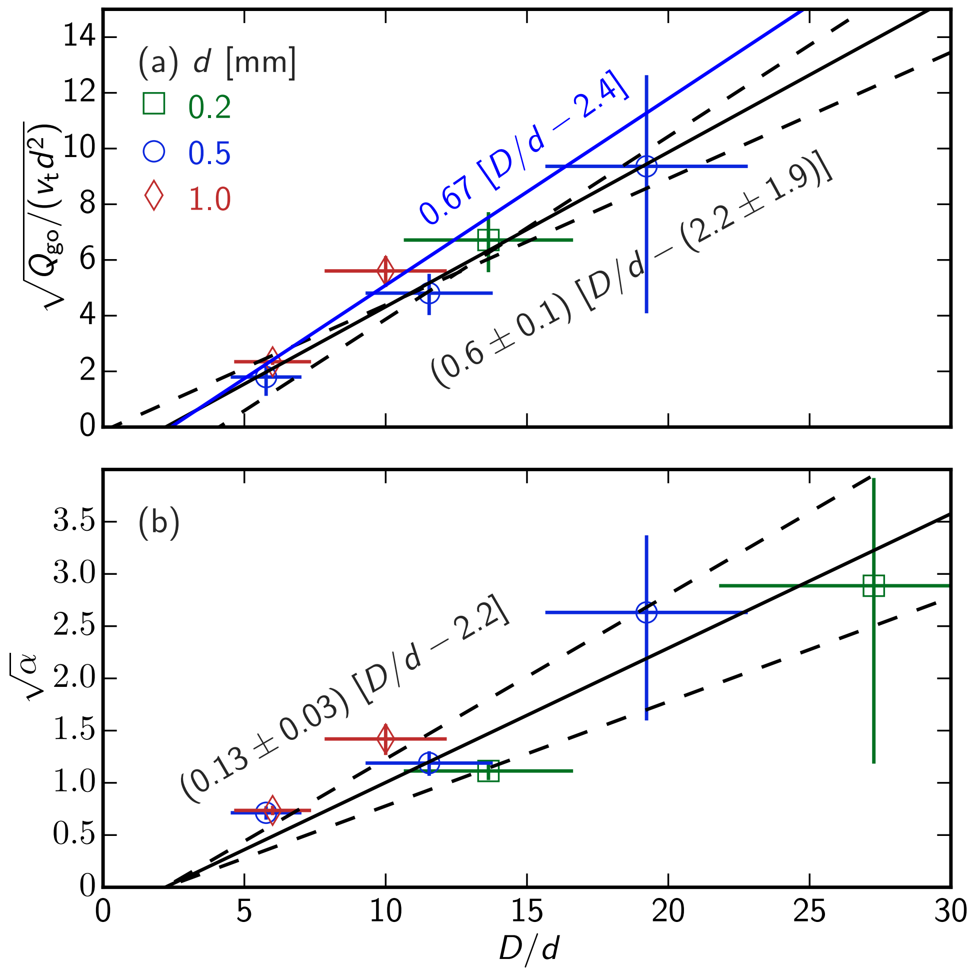

Supplementary Figure 3: Reference flow rate and grain-fluid coupling constants agree with earlier results. (a) The reference flow rate Q_{go}, at which the fluid moves down passively at the speed as the grains, as obtained using the same method as in Figure 3 in the main article for different grain and orifice diameters. The black line fit depicts the modified Beverloo equation, $Q_{\mathrm{go}} = Cv_{\mathrm{t}}d^2(D/d - k)^2$. The dimensionless fitting parameters are $C = 0.6^2 = 0.4 \pm 0.1$ and $k = 2.2 \pm 1.9$, which are close to the reported values in Wilson *et al.* [Papers in Physics **6**, 060009 (2014)]: $C_{\mathrm{w}} = 0.45$ and $k_{\mathrm{w}} = 2.4 \pm 0.1$. Here the particle diameter and its uncertainty is taken from the manufacturer. The missing data point at $D/d = 27$ is off the scale in Figure (a) and disregarded as an outlier. Figure (b) shows the excess fluid flow proportionality constant α defined by Eq. (1) of the main text. The lines represent fits with the same cutoff constant ($k = 2.2$) as in (a). In both figures, the line fits are weighted by the plotted statistical uncertainty error bars.

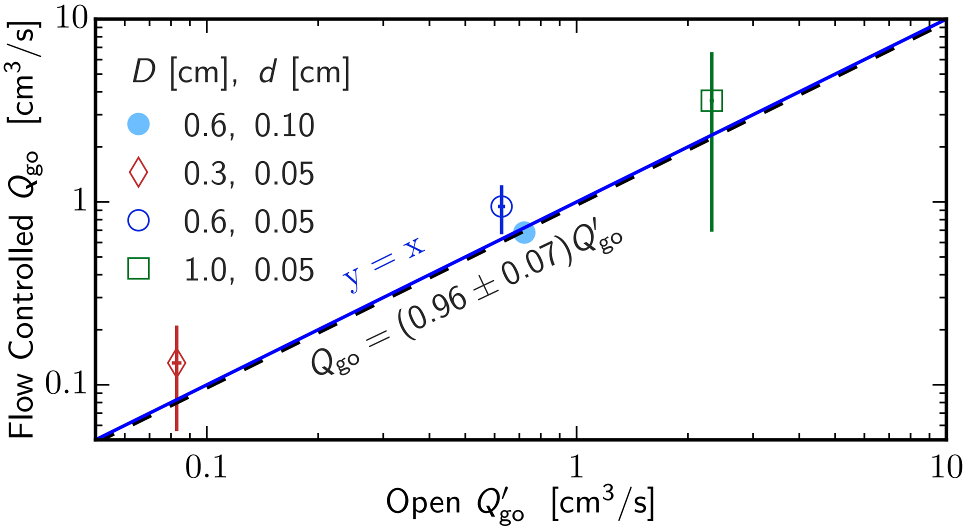

Supplementary Figure 4: Reference flow rate measured from flow controlled experiments matches the one obtained from open experiments. Reference granular discharge rates, where the fluid flows passively with the grains, from analysis of flow-controlled versus open/surge experiments. Results fall near $y = x$, showing good agreement. The dashed line is an error-weighted proportionality fit, $Q_{\rm go} = (0.96 \pm 0.07)\ Q'_{\rm go}$, consistent with $y = x$.

d [cm]	D [cm]	ΔP [Pa]	v_t [cm s^{-1}]	$\rho_f v_t^2/2$ [Pa]	$\eta v_t/d$ [Pa]	v_s [cm s^{-1}]	$\rho_f v_s^2/2$ [Pa]	$\eta v_s/d$ [Pa]
0.10	0.6	5 ± 3	15.1 ± 0.8	11 ± 1	0.15	25 ± 5	30 ± 20	0.25
0.05	0.3	15 ± 4	7.5 ± 0.4	2.8 ± 0.3	0.15	15 ± 5	11 ± 8	0.30
0.05	0.6	29 ± 7	7.5 ± 0.4	2.8 ± 0.3	0.15	30 ± 5	50 ± 20	0.60
0.05	1.0	20 ± 10	7.5 ± 0.4	2.8 ± 0.3	0.15	30 ± 5	50 ± 20	0.60

Supplementary Table 1: Speeds and pressures for various diameter grains and orifices. Here v_t is the terminal speed of isolated grains, as computed from the drag coefficient [F.A. Morrison, Data Correlation for Drag Coefficient for Sphere (www.chem.mtu.edu/˜fmorriso, accessed June 2012)]; the corresponding Reynolds numbers are 37 and 150 for 0.5 and 1.0 mm grains, respectively. The speed v_s of the stream of discharging grains is measured by video with an accuracy of 5 cm/s. These speeds translate to characteristic Bernoulli and viscous pressure scales, as tabulated. For comparison, the fitting parameter a defined by Eqs. (4,5) of the main text gives the actual pressures ΔP, in the third column, that drive the permeation flow. Note that ΔP corresponds most closely to the Bernoulli pressure $\rho_f {v_s}^2/2$, indicating that fluid flow excited under the hopper by the falling stream of grains is the source of pressure that pumps water through the grains and that -consequently- causes the surge effect.

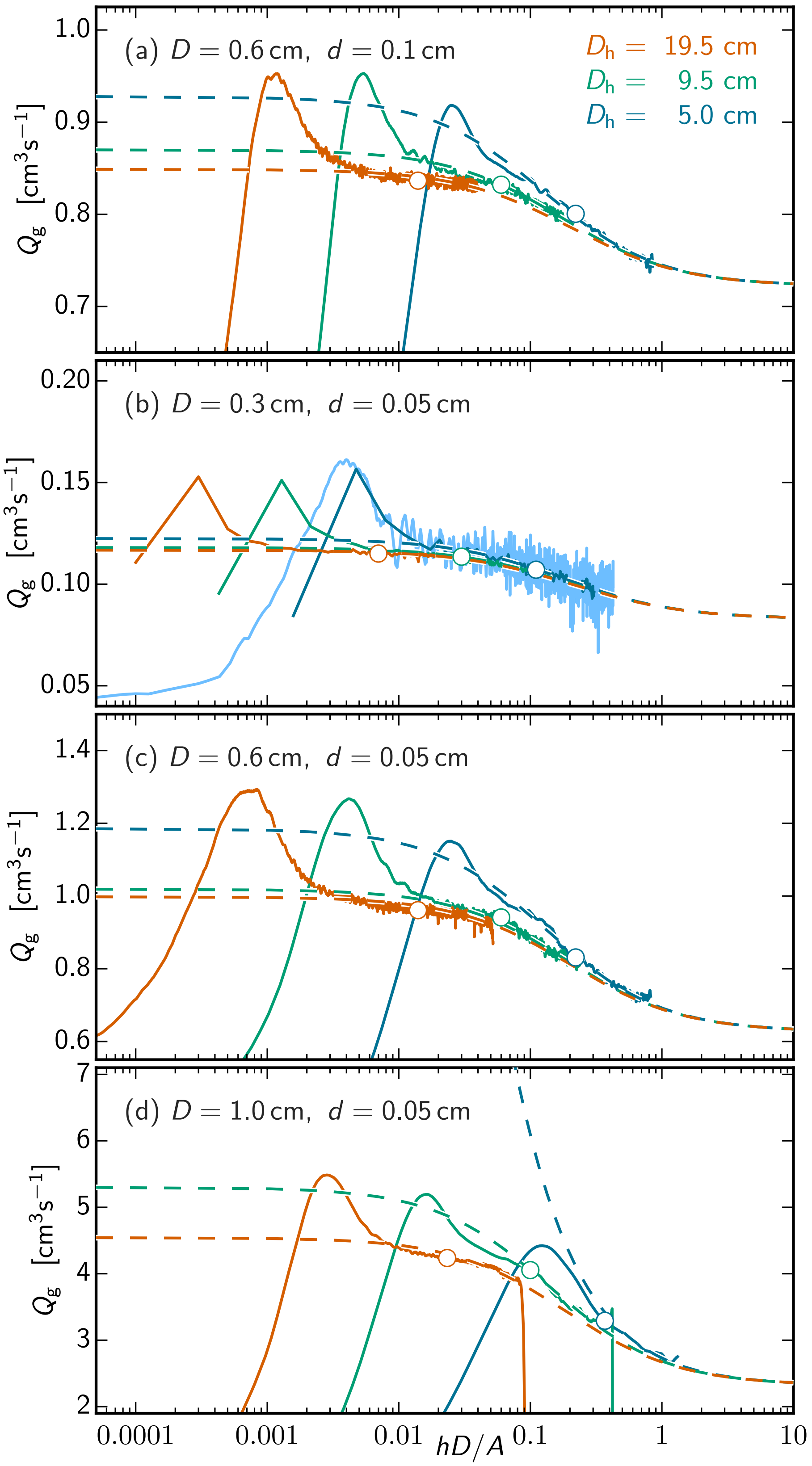

Supplementary Figure 5: Data and fits for open/surge discharge experiments with 12 combinations of grain, orifice and hopper diameters. The dashed curves represent Eq. (5) from the main text, where Q'_{go}, a and three values of b are simultaneously fitted to the each set with same d and D. The white-filled circles indicate the lower limit for the fit region, $h \geq 7$ cm. In (b) the $D = 0.3$ cm data is filtered with 2 mm windowed median. The original data is shown for $D_\mathrm{h} = 5.0$ cm case as a light blue swath. The relative noise level is higher in (b) as the flow rate is much smaller than in other cases. All analyses for $D = 0.3$ cm data use the filtered data. Properly scaled, these data sets collapse together for $h \geq \beta D$ in Fig. 4b of the main text.

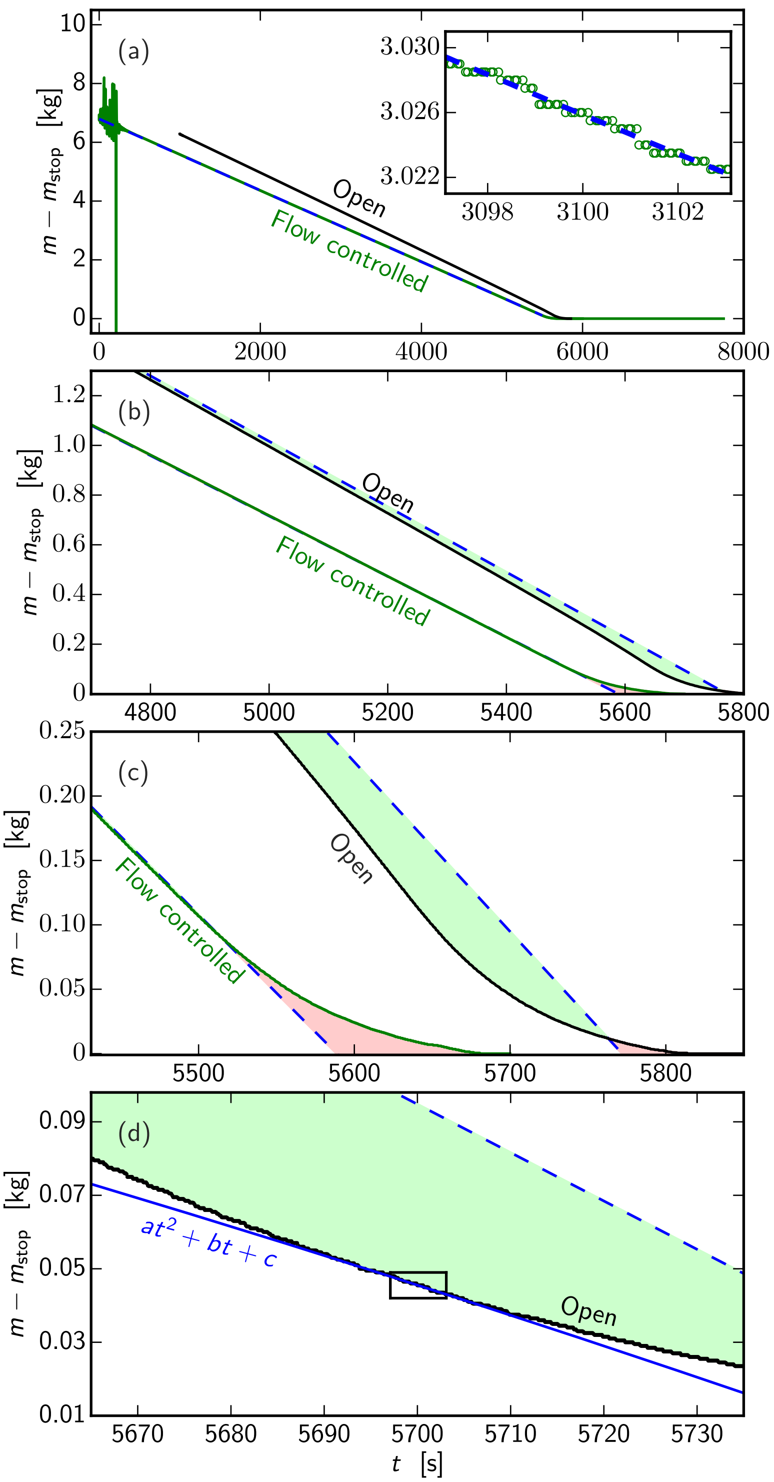

Supplementary Figure 6: The surge is seen already in the raw data. (a) The raw data from the scale is plotted with the green curve (and circles in the inset). The final weight $m_{\rm stop}$ at the end of the experiment when the grains stop flowing is subtracted from the data. At the beginning of the experiment there are oscillations due to the starting procedure. The black curve is an open experiment with the same geometry. The dashed blue line is a linear fit from $t = 1000$ s to $t = 4000$ s that, in this figure only, is considered the linear steady state. The inset is a magnification corresponding to differentiation window size with less than 1% of the entire dataset. Figures (b,c,d) are magnifications of Figure (a) with same curves and fits. The light green shaded areas highlight the increased flow rate in comparison with the linear fit. The increase in flow rate is only seen in the open case. The shaded red area at the end is the region where the hopper begins to run out of grains and the flow rate decreases. The black box in Figure (d) shows the Gaussian weighted differentiation window of $t_{\rm w} = 2\sigma = 6$ s used in the calculation of grain flow rate $Q_{\rm go}$. The solid blue line in Figure (d) is the weighted 2$^{\rm nd}$ degree polynomial fit $m - m_{\rm stop} = at^2 + bt + c$ to the data inside the black box. The derivative $\mathrm{d}m/\mathrm{d}t$ is the first-degree term b in this fit.

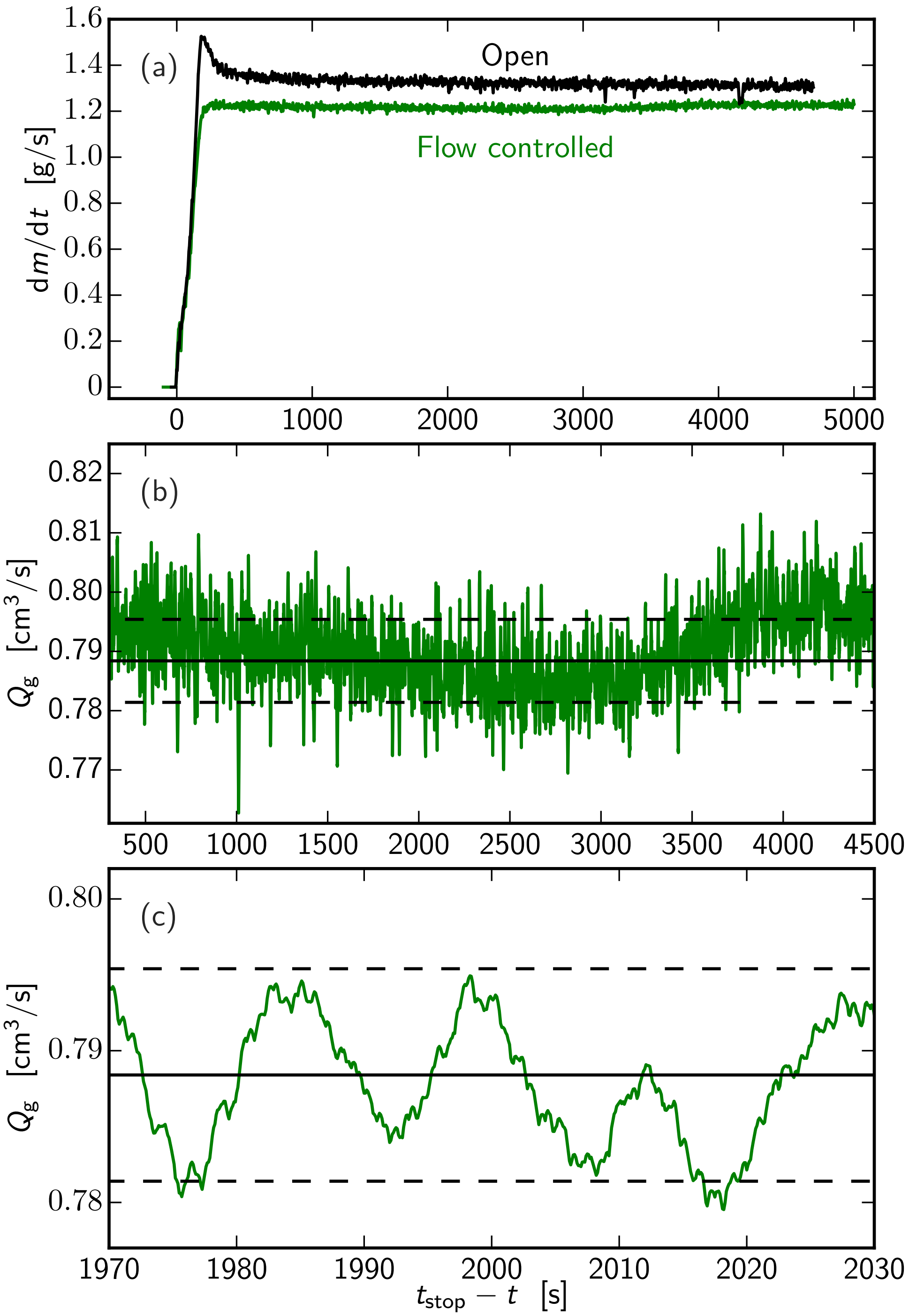

Supplementary Figure 7: The flow controlled experiment has a constant flow rate within measurement accuracy. (a) The mass flow rate $\mathrm{d}m/\mathrm{d}t$ from the windowed numerical derivative, for a flow controlled experiment (green curve) and an open experiment (black curve). Both curves are from the same data as in Supplemental Figure 6. The final stop time t_{stop} is defined as when the flow stops and corresponds to the final weight m_{stop}. (b) The green data is the volume flow rate $Q_{\mathrm{g}} = -(\rho_{\mathrm{glass}} - \rho_{\mathrm{f}})^{-1}\mathrm{d}m/\mathrm{d}t$, where the prefactor $(\rho_{\mathrm{glass}} - \rho_{\mathrm{f}})^{-1}$ takes the buoyancy of the water into account. The horizontal bars show the average flow rate (solid) and its error limits as one standard deviation from the mean. The data set is the same as in Figure 2a. (c) The data in Figure b is magnified. The oscillations in this scale are random walk with power spectrum that scales as Brownian noise exponent 2.
